# Assessment of Femoral Trochlear Groove Depth in Canine Cadavers with Normal Stifle Joints Using Ultrasonography with the Joint Positioned at 45°, 90°, and Hyperextension, and Radiography in the Skyline Projection

**DOI:** 10.3390/ani16030514

**Published:** 2026-02-06

**Authors:** Amanda Junqueira, Maria Paula Luchi da Silva Mattos, Francine Hergemoller, Thayse Meyer, Caroline Bernardo Gusmão, Rafael Kretzer Carneiro, Márcio Poletto Ferreira

**Affiliations:** 1 Department of Veterinary Medicine, Federal University of Rio Grande do Sul (UFRGS), Av. Bento Gonçalves, Agronomia, Porto Alegre 91540-000, RS, Brazil; amandamcjunqueira@gmail.com (A.J.); francine_hergemoller@hotmail.com (F.H.); meyerthayse@gmail.com (T.M.); caroline.gusmao@hotmail.com (C.B.G.); marcio.ferreira@ufrgs.br (M.P.F.); 2 Laboratory of Diagnostic Imaging, Center for Agroveterinary Sciences (CAV), Department of Veterinary Medicine, Santa Catarina State University (UDESC), Lages 88520-000, SC, Brazil; maria.mattos@edu.udesc.br

**Keywords:** diagnostic imaging, canine, orthopedics, stifle, joint, trochlear groove

## Abstract

This prospective, analytical study aimed to measure and compare trochlear groove depth using skyline radiographic projection and ultrasonography at three stifle joint angles and four distinct anatomical regions, as well as to compare these findings with ex vivo measurements obtained using a caliper under the same conditions in canine cadavers without patellar luxation. A total of 67 limbs were evaluated. Ultrasonography demonstrated greater agreement with ex vivo measurements than radiography. Ultrasonographic assessment was considered appropriate for measuring trochlear groove depth with the stifle joint in maximal extension, with the transducer positioned distal to the patella, and at 90° of flexion, with the transducer positioned proximal to the patella.

## Simple Summary

Trochleoplasty is indicated in dogs with patellar luxation and aims to deepen the trochlear groove depth. Radiography and ultrasonography may assist in the preoperative assessment of trochlear groove depth; however, both techniques have limitations related to standardization. This cadaveric study aimed to compare trochlear groove depth measured using the skyline radiographic projection and ultrasonography at different stifle joint angles and anatomical regions, as well as to compare these findings with ex vivo measurements obtained using a caliper. Ultrasonography demonstrated greater agreement with ex vivo measurements than radiography. In addition, the skyline projection showed greater variability among measurements.

## 1. Introduction

Medial patellar luxation (MPL) is an orthopedic condition frequently observed in small-breed dogs [1,2,3]. In contrast, lateral patellar luxation occurs more commonly in medium- and large-breed dogs, such as Cocker Spaniel, Flat Coat Retriever, Bassett Hound, Newfoundland, and Springer Spaniel [4] and represents an important cause of lameness and functional impairment [5,6]. Surgical treatment is indicated and aims to restore proper alignment of the quadriceps extensor mechanism [7,8,9,10,11]. Given that the femoral trochlear groove is often shallow in dogs with MPL [12], trochleoplasty is commonly included as part of the corrective procedure, with the goal of deepening the groove to accommodate 50% of the patellar thickness [13,14]. However, the decision to perform trochleoplasty is frequently made intraoperatively and remains largely subjective, relying on visual inspection after arthrotomy and the surgeon’s experience [15].

Given the difficulty in determining, during the preoperative period, the need for trochleoplasty, it is essential to establish objective methods capable of quantifying trochlear groove depth in dogs. Traditionally, this depth is measured in dogs using the skyline radiographic projection; however, the degree of stifle joint flexion may affect the accuracy of this assessment [16]. Computed tomography (CT) has also been described as a complementary tool for evaluating the trochlear groove, most commonly through measurement of the sulcus angle, in Toy Poodles, Pomeranians, and Chihuahuas [12]. Although CT allows for three-dimensional assessment, it is a high-cost modality that, similar to skyline radiography, requires sedation or general anesthesia [17]. In addition, neither imaging modality allows evaluation of the articular cartilage [18,19], which, in the authors’ opinion, may result in overestimation of trochlear groove depth.

Ultrasonography is a valuable tool for musculoskeletal evaluation in both human and veterinary medicine, as it is a non-invasive imaging modality that does not involve ionizing radiation and, in most cases, does not require sedation or general anesthesia [20,21,22,23]. Studies have investigated the femoral trochlear groove using ultrasonography in humans [24,25] and in dogs [26,27]. However, although several studies have evaluated trochlear morphology, to the best of the authors’ knowledge, no studies have compared trochlear groove depth measured by ultrasonography and skyline radiography with ex vivo assessment. The authors consider this comparison relevant, as ex vivo measurement represents the most accurate method and closely approximates intraoperative assessment, which is widely used to guide the decision of whether to perform trochleoplasty

The objectives of this study were to compare trochlear groove depth assessed by skyline radiography and ultrasonography, at three different joint angles and across four anatomical regions, with ex vivo measurements obtained using a caliper in canine cadavers without patellar luxation, in order to evaluate the applicability of these imaging methods in decision-making regarding the performance of trochleoplasty.

## 2. Materials and Methods

### 2.1. Selection and Description of Subjects

This prospective study was approved by the Animal Use Ethics Committee (CEUA) of the Federal University of Rio Grande do Sul under protocol number 36369. A total of 67 pelvic limbs from adult dogs, all mixed-breed dogs, paired and unpaired, male and female, with a mean body weight of 19.04 ± 5.76 kg and a mean age of 5.2 ± 1.8 years, and euthanized for reasons unrelated to this project, were selected. All limbs underwent clinical evaluation (cranial drawer test, tibial compression test, flexion and extension of the stifle joint, and assessment of patellar instability) and radiographic evaluation in standard orthogonal projections (craniocaudal) to ensure inclusion of only those without osteoarticular abnormalities. For each patient, kilovoltage (kV) and milliampere-seconds (mAs) were adjusted to obtain an adequate radiographic image. Stifles with degenerative joint disease, fractures, angular deformities, or open growth plates were not included in the study.

After inclusion in the study, the limbs were disarticulated at the coxofemoral joint and stored in a freezer at −26 °C for 48 h. After this period, the limbs were thawed at room temperature. Following thawing, the limbs were clipped in the femorotibiopatellar joint region and submitted to radiographic examination in skyline projection, followed by ultrasonographic evaluation and subsequent skeletonization.

### 2.2. Radiographic Examination

All limbs were radiographed in craniocaudal, mediolateral at 90° flexion, and skyline at 45° flexion projections. All imaging procedures were performed using a 1-inch round metallic radiographic marker positioned at the level of the femorotibiopatellar joint, and joint angulations were adjusted with the aid of a digital goniometer. A horizontal x-ray beam was used to obtain the skyline images.

**Figure 1:**
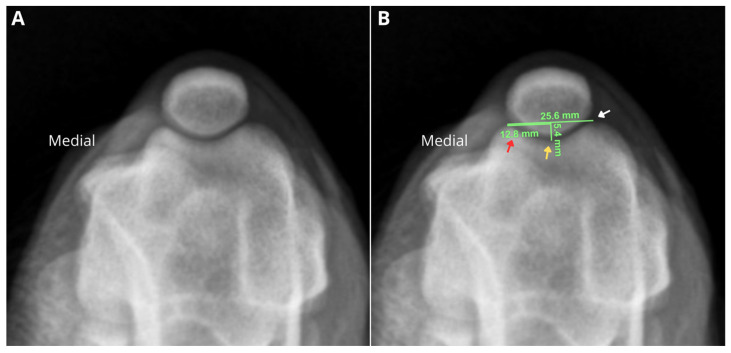
Radiographic evaluation of the right pelvic limb stifle joint of a canine cadaver in the tangential (craniodistal–cranioproximal oblique) projection at 45° of flexion to assess trochlear groove depth. (**A**). In (**B**), a line tangent to the femoral trochlear ridges is marked, showing a value of 25.6 mm (white arrow). Next, a line parallel to the first was drawn to determine the midpoint of the trochlear groove (12.8 mm) (red arrow), and from this point, a line perpendicular to the initial line was extended to the base of the trochlear groove (yellow arrow), obtaining the trochlear depth measurement (5.4 mm).

The skyline radiographic projection was used to measure the trochlear groove depth (TGD). Craniocaudal and mediolateral projections at 90° flexion were used to exclude orthopedic diseases such as osteoarthritis, deformities, and others. To calculate the TGD, a line was drawn tangent to the femoral condyles. Then, a second line perpendicular to the first was drawn at the midpoint, extending to the femoral trochlear groove. The length of this second line corresponded to the trochlear depth measurement (Figure 1). Virtual Preoperative Orthopaedic Planning Tool (VPOP^®^, Version 3.0.10) was used for the calculation of the trochlear groove depth.

Radiographic examinations were performed using a conventional x-ray unit (Siemens^®^ RG150/100gl). Images were acquired on 35 cm × 43 cm AGFA plates and subsequently digitized using an AGFA computed radiography system (model CR-30). All images were then evaluated on a workstation equipped with DICOM viewing software (Version 2025.1) by a single evaluator (MPF).

### 2.3. Ultrasonographic Examination

Ultrasonographic examinations were performed immediately after the radiographic assessments. All examinations were conducted using a Myndray Z6vet unit (Myndray^®^, Shenzhen, China) with a linear transducer operating at a frequency range of 8–10 MHz. Ultrasound coupling gel was applied throughout the examination. Gain, depth, and focal settings were adjusted as needed for each stifle.

Ultrasonographic examination was performed with the joint positioned at three different angles and assessed in four distinct regions, aiming to evaluate the trochlear groove depth at different portions of the trochlea. For joint goniometry adjustments, a 200 mm digital goniometer was used on the lateral aspect of the limb, with the stifle joint as the central reference and the greater trochanter and tarsus as the proximal and distal reference points, respectively.

**Figure 2:**
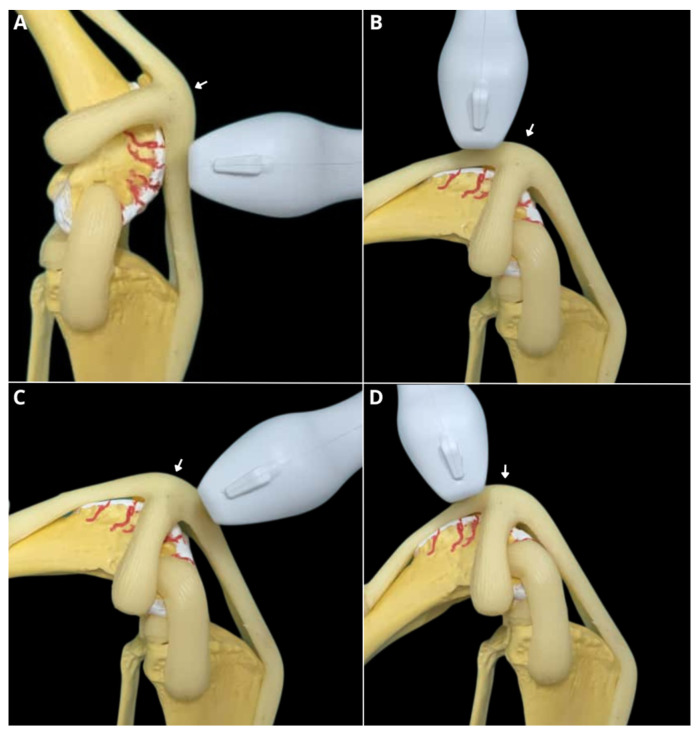
Ultrasonographic evaluation of a canine stifle joint anatomical specimen for measurement of trochlear groove depth. Depth measurement in maximum joint extension (140°) with the ultrasound transducer positioned distal to the patella (white arrow) (**A**). Depth measurement at 90° joint flexion with the transducer positioned proximal to the patella (white arrow) (**B**). Depth measurement at 90° joint flexion with the transducer positioned distal to the patella (white arrow) (**C**). Depth measurement at 45° joint flexion with the transducer positioned proximal to the patella (white arrow) (**D**).

**Figure 3:**
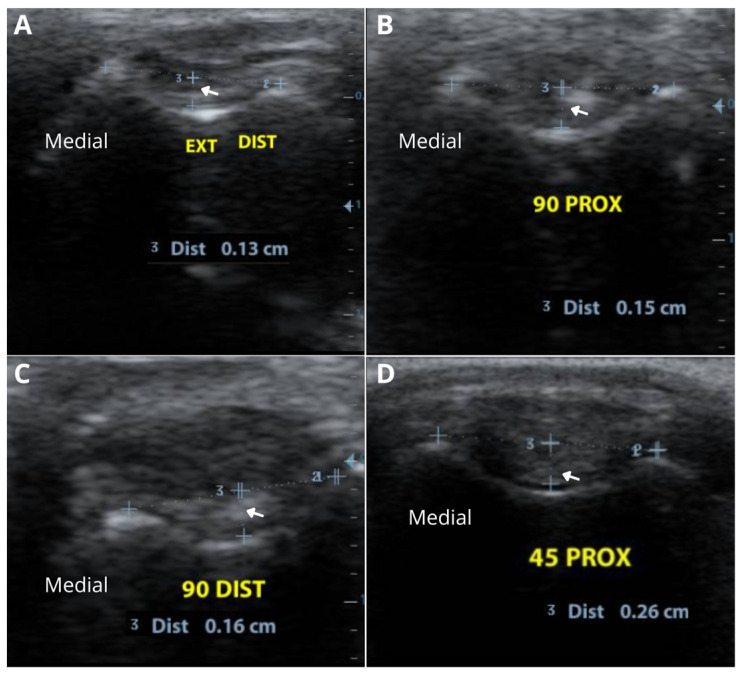
Ultrasonographic evaluation of a canine stifle joint for measurement of trochlear groove depth. (**A**) Depth measurement in maximum joint extension (140°) with the transducer positioned distal to the patella, showing a trochlear groove depth of 0.13 cm (White arrow, Dist 3). (**B**) Depth measurement at 90° flexion with the transducer positioned proximal to the patella, showing a trochlear groove depth of 0.15 cm (White arrow, Dist 3). (**C**) Depth measurement at 90° flexion with the transducer positioned distal to the patella, showing a trochlear groove depth of 0.16 mm (White arrow, Dist 3). (**D**) Depth measurement at 45° flexion with the transducer positioned proximal to the patella, showing a trochlear groove depth of 0.26 cm (White arrow, Dist 3).

The transducer was always positioned perpendicular and transverse to the trochlear groove. Initially, the limb was held in full extension, and the transducer was placed distal to the patella. Then, with the joint flexed at 90°, the transducer was placed both proximal and distal to the patella. Finally, with the joint flexed at 45°, the transducer was positioned proximal to the patella (Figure 2 and Figure 3).

The depth of the femoral trochlear groove was assessed by drawing a line connecting the highest cartilaginous points of the medial and lateral trochlear ridges, and then drawing a perpendicular line to the deepest cartilaginous surface of the trochlear groove (Figure 3).

### 2.4. Arthrotomy and Measurement with a Caliper

After all ultrasonographic measurements were completed, stifle arthrotomies were performed to expose the trochlear groove for in situ measurement with a caliper. A digital caliper (Davidson^®^, Berlin, Germany) was used for all assessments. Goniometry adjustments were performed as described for the ultrasonographic evaluation.

**Figure 4:**
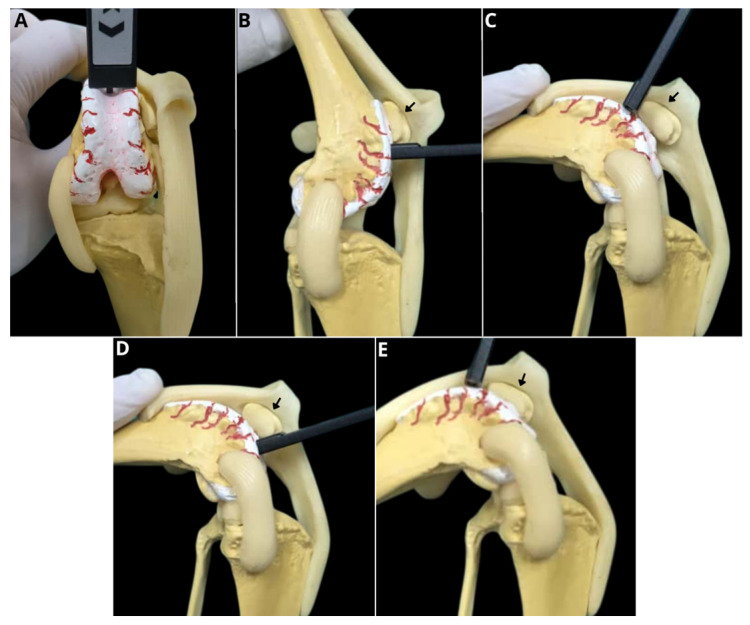
Assessment of trochlear depth using a digital caliper in an anatomical canine model. Craniocaudal view of the caliper with medial displacement of the patellar ligament and patella (**A**). Mediolateral view. Positioning of the caliper distal to the patella with the joint in hyperextension (140°) (black arrow) (**B**). Mediolateral view. Positioning of the caliper proximal to the patella with the joint flexed at 90° (black arrow) (**C**). Mediolateral view. Positioning of the caliper distal to the patella with the joint flexed at 90° (black arrow) (**D**). Mediolateral view. Positioning of the caliper proximal to the patella with the joint flexed at 45° (black arrow) (**E**).

For the arthrotomy, the limb was positioned in medial recumbency, and a longitudinal skin incision was made lateral to the stifle joint, allowing medial luxation of the patella and visualization of the articular structures (Figure 4).

### 2.5. Data Recording and Statistical Analysis

All measurements were performed after completing the sample collection in order to allow randomization of the images and to prevent the same limb from being evaluated simultaneously by the reviewers. All radiographic examinations were positioned, assessed, and measured by a single evaluator, a veterinary specialist with 15 years of experience in the field (MPF).

Ultrasonographic evaluations of the trochlear grooves were performed by a veterinary specialist in ultrassonography (AJ), who underwent six months of prior training before the study commenced. The evaluations were conducted blindly, with the operator blinded to the radiographic measurements. Ex vivo measurements were performed by a trained operator (RKC), who was also blinded to the radiographic and ultrasonographic results.

Data were entered into Excel spreadsheets and subsequently exported to SPSS version 20.0 for statistical analysis. Categorical variables were described as frequencies and percentages. Quantitative variables were described using mean and standard deviation. Femoral trochlear measurements obtained by ultrasonography and radiography were compared to anatomical measurements using the paired t-test. The Bland–Altman method was employed to assess agreement between the methods. A significance level of *p* < 0.05 was considered for all comparisons.

## 3. Results

A total of 90 pelvic limbs from canine cadavers were evaluated, but 23 were excluded due to the presence of osteoarthritis (*n* = 5), immature animals with open growth plates (*n* = 12), and patellar luxation (*n* = 6). Thus, only 67 limbs were included in the study.

### 3.1. Radiographic Evaluation

Radiographic examination enabled joint assessment and assisted in the selection of stifles for the study. The mean trochlear groove depth, measured in the 67 limbs using the tangential projection, is presented in Table 1.

### 3.2. Ultrasonographic Evaluation

The trochlear groove depth at the proximal region of the patella, with the joint flexed at 45°, was 0.22 ± 0.06 cm. At the proximal patellar evaluation with the joint flexed at 90°, a depth of 0.217 ± 0.070 cm was observed. The distal patellar measurement at 90° flexion showed a depth of 0.341 ± 0.207 cm, and at full extension, it was 0.181 ± 0.049 cm (Table 1). The trochlear groove was easily identified and measured in most assessments, except at the 90° angle with the transducer positioned distal to the patella. In this evaluation, even with the transducer maintained perpendicular to the trochlea, the base of the groove was not fully visible in some cases due to patellar overlap.

### 3.3. Ex Vivo Evaluation

Ex vivo measurements were used as the gold standard to compare radiographic and ultrasonographic measurements. No difficulties were encountered during the arthrotomy. Marking the region of interest with a permanent marker facilitated identification and ensured that the ex vivo caliper measurements of the trochlear groove were taken at the same locations as the ultrasonographic assessments. The trochlear groove depth at the proximal region of the patella, with the joint flexed at 45°, was 0.189 ± 0.062 cm. At the proximal patellar evaluation with the joint flexed at 90°, a depth of 0.216 ± 0.078 cm was observed. The distal patellar measurement at 90° flexion showed a depth of 0.203 ± 0.081 cm, and at full extension, it was 0.176 ± 0.07 cm (Table 1).

### 3.4. Discriminant Analysis

When comparing radiographic measurements obtained from the skyline projection with ex vivo measurements, no statistically significant difference was observed only for trochlear groove depth at 90° proximal flexion (*p* = 0.12) (Table 2). Regarding ultrasonographic measurements, only assessments performed at full extension (*p* = 0.39) and at 90° proximal flexion (*p* = 0.80) showed no statistically significant differences when compared with the ex vivo evaluation (Table 3). When comparing the skyline projection results with the ultrasonographic evaluations, similarity was observed only at 45° flexion with the transducer positioned proximal to the patella (*p* = 0.22) (Table 4). The remaining *p* values among the assessments are detailed in Table 2.

## 4. Discussion

After data analysis, our initial hypotheses were partially rejected. Although some ultrasonographic measurements were consistent with ex vivo evaluations, this agreement was not observed across all assessed regions. Furthermore, the trochlear groove depth measured via the radiographic skyline projection differed from the ex vivo measurement at 45° flexion. This study provides relevant contributions regarding the reliability of ultrasonography and radiography in evaluating trochlear morphology by directly comparing these techniques to anatomical measurements obtained from canine cadavers.

The decision to perform trochleoplasty remains a challenge in the surgical planning of patellar luxation, even with the aid of imaging methods. Although trochlear groove depth can be measured using radiographic skyline projection, ultrasonography, or computed tomography, there is still no consensus on the optimal limb angulation during these examinations to evaluate the deepest region of the trochlea, resulting in highly individualized assessment approaches [14,17,18,28,29]. Additionally, the authors believe that the absence of cartilage evaluation in radiographic examination tends to yield trochlear groove depth values higher than those obtained ex vivo, erroneously suggesting deeper trochlear grooves.

The mean trochlear groove depth measured radiographically by the skyline projection (0.228 ± 0.062 cm) was greater than most ex vivo measurements, except for the evaluation performed at 90° proximal flexion. Thus, the authors believe that this traditional approach may not accurately reflect the true depth of the trochlear groove, especially when cartilage is not considered [30]. Moreover, despite standardizing the limb at 45° flexion for the skyline projection, the incidence of the x-ray beam did not align perfectly with the angulation of the trochlear groove, which may have contributed to the discrepancies observed relative to ultrasonographic and ex vivo values.

Only ultrasonographic measurements performed at full extension and at 90° proximal flexion of the trochlear region were similar to the ex vivo measurements. The authors believe that joint positioning during ultrasonographic examination may directly influence the exposure and image definition of the trochlear groove. At full extension and 90° flexion, the orientation of the trochlea appears to favor a more tangential alignment of the ultrasound beam relative to the bone surface, improving acoustic windowing and allowing for more precise delineation of anatomical landmarks. Furthermore, at these angles, patellar interference with groove visualization is minimized, particularly in the proximal portion.

In this study, ultrasonographic measurements showed better agreement with ex vivo values, suggesting that this modality can accurately delineate the depth of the trochlear groove by considering the contour of the articular cartilage [26], thus providing more reliable results and representing a feasible tool for decision-making regarding whether or not to perform trochleoplasty. Interestingly, the poorest ultrasonographic visualizations were observed at 90° flexion distal to the patella and at 45° proximal to the patella. This finding is possibly due to the patella overlapping the groove at these angles, hindering precise delineation. These results underscore the importance of appropriate joint angulation and transducer orientation to ensure optimal anatomical exposure of the trochlear groove while minimizing patellar interference.

This study has several limitations, notably the difficulty in obtaining a homogeneous sample (weight, sex, breed, and age), which may have introduced natural anatomical variability in trochlear groove morphology, affecting the values obtained for comparison between imaging methods and ex vivo measurements. Another limitation is the sample size, which, although statistically sufficient to demonstrate significant results, could be increased to further enhance the reliability of the findings. Additionally, advanced imaging techniques, such as computed tomography, were not used to more accurately assess potential morphological alterations in the limbs under study. Despite the use of a goniometer to standardize stifle joint angulation during image acquisition, slight joint movements may have occurred, as the limbs were not secured with external stabilization devices, potentially influencing the measurements, particularly in ultrasonographic evaluations. Finally, it is recognized that ultrasonography is an operator-dependent technique, and other operators may obtain results that differ from those achieved in this study.

## 5. Conclusions

Ultrasonography is a reliable method when performed at 90° proximal flexion and maximum extension for measuring trochlear groove depth, demonstrating better agreement with anatomical measurements than the traditional radiographic skyline projection. Trochlear groove depth was 0.217 ± 0.07 cm at 90° proximal flexion and 0.181 ± 0.04 cm at maximum extension. Ultrasonography may be recommended as a diagnostic tool for surgical planning of patellar luxation.

## Figures and Tables

**Table 1:** Measurements of the trochlear groove depth using skyline radiography, ultrasonography, and ex vivo caliper assessment at four joint angles.

	Radiography	Ultrasonography	Ex Vivo
Maximum extension	-	0.181 ± 0.04	0.176 ± 0.02
90° Flexion—Proximal	-	0.217 ± 0.07	0.216 ± 0.07
90° Flexion—Distal	-	0.341 ± 0.20	0.203 ± 0.08
45° Flexion—Proximal	-	0.220 ± 0.06	0.189 ± 0.06
Skyline Projection	0.228 ± 0.06	-	-

Data are presented as mean ± standard deviation (cm).

**Table 2:** Comparison between skyline radiographic and ex vivo measurements at four joint angles of trochlear groove depth.

	Radiography	Ex Vivo	*p* < 0.05
Maximum extension	-	0.176 ± 0.07	<0.001
90° Flexion—Proximal	-	0.216 ± 0.07	0.12 *
90° Flexion—Distal	-	0.203 ± 0.08	0.02
45° Flexion—Proximal	-	0.189 ± 0.06	<0.001
Skyline Projection	0.228 ± 0.06	-	-

Data are presented as mean ± standard deviation (cm). * Results with no statistically significant difference. The *p*-values were obtained from the comparison between radiographic and ex vivo results.

**Table 3:** Comparison between ultrasonographic and ex vivo measurements at four joint angles of trochlear groove depth.

	Ultrasonography	Ex Vivo	*p* < 0.05
Maximum extension	0.181 ± 0.04	0.176 ± 0.07	0.39 *
90° Flexion—Proximal	0.217 ± 0.07	0.216 ± 0.07	0.80 *
90° Flexion—Distal	0.341 ± 0.20	0.203 ± 0.08	<0.001
45° Flexion—Proximal	0.220 ± 0.06	0.189 ± 0.06	<0.001

Data are presented as mean ± standard deviation (cm). * Results with no statistically significant difference. The *p*-values were obtained from the comparison between ultrasonographic and ex vivo results.

**Table 4:** Comparison between ultrasonographic measurements at four joint angles and skyline radiographic measurements of trochlear groove depth.

	Radiography	Ultrasonography	*p* < 0.05
Maximum extension	-	0.181 ± 0.04	<0.001
90° Flexion—Proximal	-	0.217 ± 0.07	0.07 *
90° Flexion—Distal	-	0.341 ± 0.20	<0.001
45° Flexion—Proximal	-	0.220 ± 0.06	0.22 *
Skyline Projection	0.228 ± 0.06	-	-

Data are presented as mean ± standard deviation (cm). * Results with no statistically significant difference. The *p*-values were obtained from the comparison between radiographic and ultrasonographic results.

## Data Availability

The raw data supporting the conclusions of this article will be made available by the authors on request.
